# Diagnostic dilemma: a large pancreatic lipoma initially misdiagnosed as liposarcoma on CT imaging

**DOI:** 10.1093/bjrcr/uaag013

**Published:** 2026-04-20

**Authors:** Saeed Mohammadzadeh, Alisa Mohebbi, Afshin Mohammadi, Ata Abbasi, Rahim Mahmodlou

**Affiliations:** Universal Scientific Education and Research Network (USERN), Tehran, 1931973111, Iran; Department of Radiology, Tehran University of Medical Sciences, Imam Khomeini Hospital, Tehran, 1419733141, Iran; Universal Scientific Education and Research Network (USERN), Tehran, 1931973111, Iran; Department of Radiology, Tehran University of Medical Sciences, Imam Khomeini Hospital, Tehran, 1419733141, Iran; Department of Radiology, Faculty of Medicine, Urmia University of Medical Science, Urmia, 5756115111, Iran; Cellular and Molecular Research Center, Cellular and Molecular Medicine Research Institute, Urmia University of Medical Sciences, Urmia, 5756115111, Iran; Department of Pathology, Faculty of Medicine, Urmia University of Medical Sciences, Urmia, 5756115111, Iran; Department of Surgery Imam Khomeini Hospital, Urmia University of Medical Sciences, Urmia, 5756115111, Iran

**Keywords:** pancreatic lipoma, pancreatic liposarcomas, Whipple surgery, CT, case report

## Abstract

Pancreatic lipomas are exceptionally rare benign mesenchymal tumors that may closely resemble well-differentiated liposarcomas on imaging, particularly when large or atypical. We report the case of a 60-year-old man who presented with abdominal pain, nausea, and vomiting, and was found to have a large fat-containing mass in the pancreatic head on computed tomography. Imaging characteristics—including size, mild heterogeneity, and a cystic component—raised strong suspicion for a well-differentiated liposarcoma. Due to diagnostic uncertainty and potential oncologic risk, the patient underwent a pancreaticoduodenectomy (Whipple procedure). Histopathological evaluation revealed a benign pancreatic lipoma composed of mature adipocytes without atypia or lipoblasts. This case highlights the diagnostic limitations of imaging in differentiating benign from malignant fat-containing pancreatic lesions and underscores the essential role of histopathology for definitive diagnosis. Surgical resection remains crucial when imaging is inconclusive or when malignancy cannot be confidently excluded.

Key Clinical Message:
Pancreatic lipomas are extremely rare, benign mesenchymal tumors that can mimic liposarcomas on imaging. A 60-year-old man with a large pancreatic mass (suspected liposarcoma on CT) underwent a Whipple procedure, revealing a benign lipoma. Surgical resection is important for suspicious masses, as histopathology is needed for definitive diagnosis and management.

## Introduction

Pancreatic lipomas are exceedingly rare, benign mesenchymal tumors that constitute a small fraction of all pancreatic tumors, with an estimated incidence of 0.012% in patients undergoing routine cross-sectional imaging.^1^ Among retroperitoneal organs, the pancreas is an extremely rare region for primary liposarcoma, with fewer than 50 cases reported in the literature.^2^ The etiology of pancreatic liposarcoma is unknown, although trauma or radiation exposure have been suggested.^2^ These tumors are typically easy to identify on imaging, particularly with computed tomography (CT), due to their characteristic features such as homogeneity and low density.^3^^,^^4^ However, differentiating them from well-differentiated liposarcomas can be challenging without definitive histopathological confirmation, as both may present with similar radiographic appearances.^5^

Pancreatic lipomas are generally asymptomatic and incidentally discovered during imaging for unrelated reasons.^6^ They are usually well-circumscribed, homogeneous, and show no contrast enhancement on CT scans, with attenuation values ranging from −120 to −30 Hounsfield units (HU).^3^ Despite these characteristic imaging features, large pancreatic lipomas may pose diagnostic challenges due to their size and potential heterogeneity, which can mimic the appearance of well-differentiated liposarcomas.^5^ Liposarcomas, although extremely rare in the pancreas, are generally larger, more heterogeneous, and may exhibit features such as thick septa, nodular or non-adipose components, and indistinct borders.^5^ Given the rarity of pancreatic liposarcomas, with only a few reported cases, distinguishing between these two entities is crucial for appropriate management.

This case report presents an unusual instance of a large pancreatic lipoma mimicking a well-differentiated liposarcoma on CT imaging, highlighting the diagnostic challenges radiologists and surgeons face. The importance of histopathological confirmation in such cases is underscored, as it ensures accurate diagnosis and guides definitive management.

## Case presentation

A 60-year-old Middle Eastern man presented to our hospital with a 10-day history of intermittent abdominal pain and new episodes of nausea and vomiting in the past few days. The pain was described as dull and located in the upper left abdominal quadrant, occasionally radiating to the back. The patient reported no significant weight loss or changes in bowel habits. His medical history included benign prostatic hyperplasia (BPH), managed with tamsulosin 0.4 mg, with no prior history of abdominal surgeries or malignancies. There was also no significant family history of note. On physical examination, the patient appeared thin, with the only notable finding being tenderness in the upper left abdominal quadrant and a sense of fullness in the epigastric region. Laboratory tests, including complete blood count, electrolytes, liver function tests, and pancreatic enzymes, were unremarkable except for a fasting blood sugar of 117 mg/dL.

### Imaging studies

An abdominal ultrasound examination identified a well-defined, solid, hyperechoic mass within the pancreas, measuring 10 × 8 cm^2^. The lesion exhibited no evidence of calcification, cystic components, or metastatic involvement. Additionally, there was no encasement of the superior mesenteric artery (SMA) or superior mesenteric vein (SMV).

**Figure 1:**
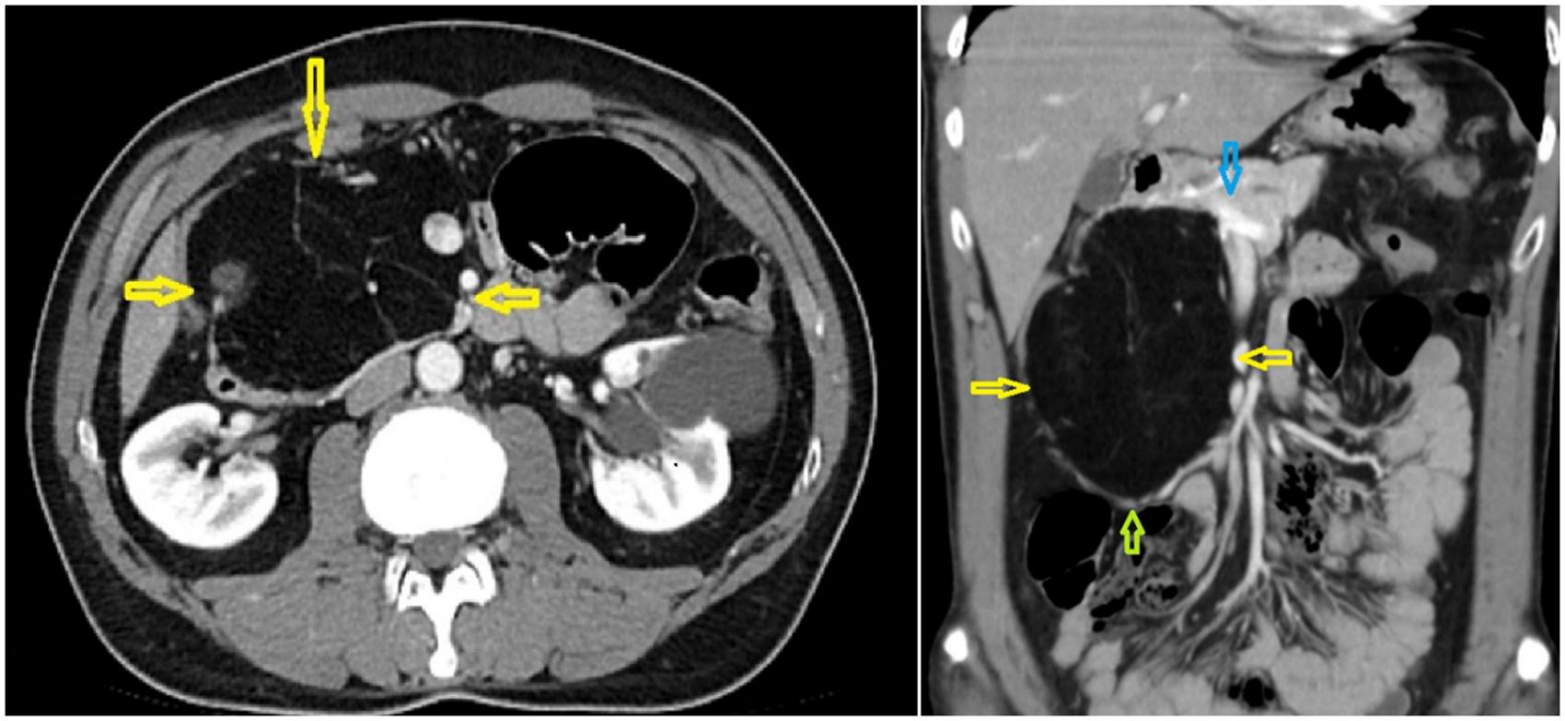
Contrast-enhanced axial and coronal CT images of a 60-year-old man with abdominal pain and vomiting demonstrate a well-defined, homogeneous, fat-attenuation mass centered in the pancreatic head. The lesion is most suspicious for a liposarcoma, supported primarily by its large size, which is a typical feature of well-differentiated liposarcomas. However, the imaging features including homogeneous macroscopic fat composition, lack of internal soft tissue nodules or post-contrast enhancement, and smooth interface with the pancreatic parenchyma are compatible with both pancreatic lipoma and well differentiated liposarcoma. Yellow arrows denote the sharply marginated, encapsulated fat-density mass in the pancreatic head. The blue arrow indicates the pancreas. The green arrow shows adjacent small-bowel loops displaced by mass effect.

For further evaluation, the patient underwent a contrast-enhanced CT examination. The CT revealed a well-defined, lipid-rich mass measuring 120 × 75 mm in the head of the pancreas, accompanied by mild soft tissue swelling and mild pancreatic duct dilation. There was no SMA or SMV involvement. A 15 × 15 mm cystic component was also observed within the mass. The mean HU of the mass was -140, consistent with a fat-containing lesion. There was no evidence of biliary duct dilation or lymphadenopathy during the CT examination (Figure 1).

Given the large size of the mass and its imaging characteristics, which raised suspicion of a liposarcoma, surgical intervention was recommended. Despite the possibility of the lesion being benign, the decision to proceed with the Whipple surgery was made due to the potential risks and diagnostic uncertainty associated with its characteristics.

### Surgery

A midline incision was performed, and during exploratory laparotomy, a large retroperitoneal mass was identified. The mass had displaced the ascending colon, transverse colon, and small bowel anteriorly. The ascending colon was mobilized, and the right ureter was identified. The viscera were rotated medially, and the mass was sharply dissected from the mesentery of the small bowel, retroperitoneum, and superior mesenteric artery and vein. Upon proximal dissection, the mass was determined to originate from the pancreatic head, completely replacing it. Further evaluation revealed that the tumor could not be resected without removing the pancreatic head. Given the suspicion of liposarcoma, a decision was made to perform a pancreaticoduodenectomy (Whipple procedure), and the excised specimen was sent to the pathology department for further workup.

### Pathological findings

**Figure 2:**
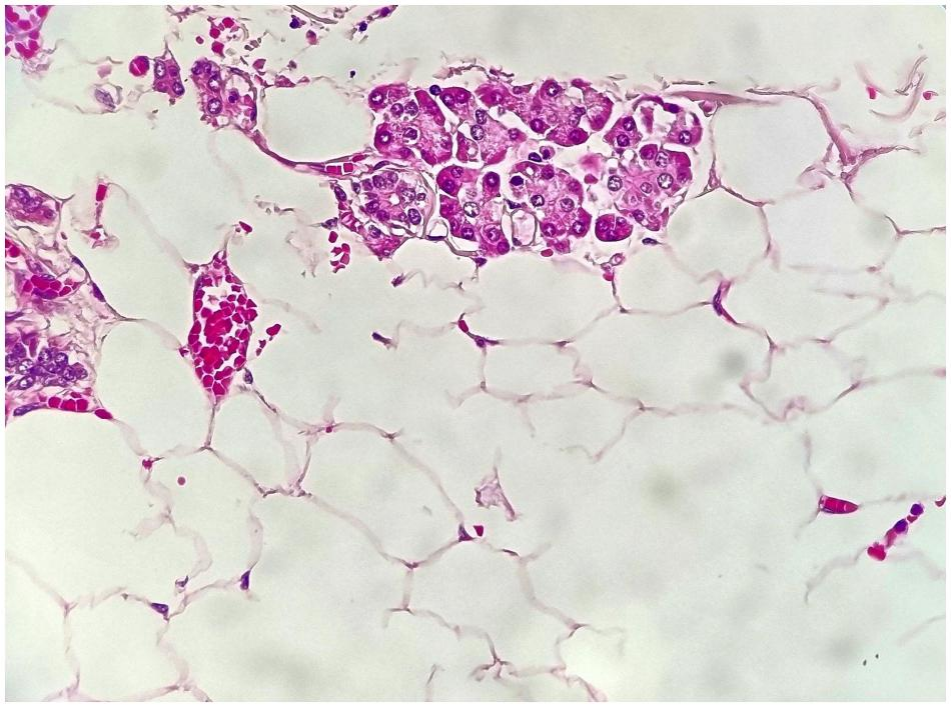
Histopathological examination of a pancreatic head biopsy from a 60-year-old man presenting with abdominal pain and vomiting reveals mature adipocytes (well-differentiated fat cells) with single large lipid vacuoles and signet-ring appearance, arranged in lobules separated by thin fibrous septa. The specimen shows no cellular atypia, mitotic activity, or lipoblasts (immature fat cells with multiple lipid droplets), ruling out liposarcoma. These microscopic features confirmed the benign lipoma diagnosis.

Gross examination of the resected specimen revealed a pancreatic mass accompanied by the duodenum, measuring 15 × 3 cm, and the pancreas itself, measuring 9 × 4 × 2.5 cm. Within the pancreas, an encapsulated mass measuring 16 × 10 × 7 cm was identified. Cut sections of the mass displayed a homogeneous yellow coloration, consistent with a lipomatous lesion. All surgical margins were intact. Microscopic examination confirmed the presence of mature adipocytes without any atypical cells or lipoblasts, consistent with a benign lipoma (Figure 2). There were no features suggestive of malignancy, such as cellular atypia, mitotic activity, or invasion into surrounding tissues. The definitive diagnosis of a benign pancreatic lipoma was thus confirmed through histopathological examination.

### Postoperative course

Postoperatively, the patient experienced hyperamylasemia, which was managed appropriately. Oral fluids were initiated on the fifth day. The patient was discharged on the seventh postoperative day with a peritoneal drainage tube in place. Two weeks after surgery, the sutures were removed. Regular follow-up appointments were scheduled every three months in the general surgery department for ongoing monitoring and care. At the time of writing this case report, nine months postoperatively, the patient remains asymptomatic with no complications.

## Discussion

Differentiating between benign lipomas and malignant fat-containing tumors, such as well-differentiated liposarcomas, can be challenging based on imaging alone. On CT, lipomas typically appear as smooth, fat-density masses separated from the pancreatic parenchyma by a thin fibrous membrane. A lipoma is typically characterized by well-defined margins, homogeneous composition, and density consistent with normal adipose tissue. It is noninvasive, clinically stable, and generally asymptomatic.^6^ In contrast, liposarcomas may exhibit heterogeneous features, including thick septa, nodular or non-adipose components, and irregular margins. However, these features are not always definitive, particularly in large or atypical lesions.^7^

To the best of our knowledge, after the Xiao et al.^5^ study, our case is the second report of a mass initially suspected to be a well-differentiated liposarcoma but ultimately confirmed as a pancreatic lipoma upon histopathological diagnosis. In our case, the lesion’s size (120 × 75 mm) and the presence of a cystic component raised suspicion for malignancy despite the predominantly fatty composition (mean HU: -140). These findings underscore the limitations of imaging in definitively distinguishing between benign and malignant fat-containing lesions, particularly in atypical presentations. Histopathological examination remains the gold standard for diagnosing pancreatic lipomas and differentiating them from liposarcomas. In our case, the gross and microscopic findings of mature adipocytes without cellular atypia or lipoblasts confirmed the benign nature of the lesion, definitively ruling out liposarcoma. In this case, preoperative biopsy (percutaneous, endoscopic, or laparoscopic) was considered but not pursued because, for a large, heterogeneous, fat-containing mass in the pancreatic head, sampling error and underestimation of a well-differentiated liposarcoma remain significant concerns, and a negative or non-diagnostic result would not have safely excluded malignancy. Our multidisciplinary team (radiology, surgery, pathology) elected to proceed directly to pancreaticoduodenectomy based on imaging appearance, lesion size and location, symptoms, and perceived oncologic risk.^8^

## Conclusion

This case report highlights the diagnostic and management challenges of large pancreatic lipomas. It underscores the importance of a multidisciplinary approach involving radiologists, surgeons, and pathologists in evaluating and treating these rare lesions. While imaging modalities fail to exclude malignancy, definitive diagnosis requires histopathological examination. Surgical intervention remains appropriate when diagnostic uncertainty exists or when lesions are symptomatic or large, ensuring both diagnostic clarity and proper treatment.

## Learning points

- Pancreatic lipomas can closely mimic malignant tumors on imaging, leading to diagnostic uncertainty.
- Imaging alone, including CT, may not reliably distinguish between benign and malignant pancreatic lesions.
- Histopathology remains the definitive method for diagnosing pancreatic masses.
- Surgical resection is important when a mass is symptomatic or cannot be confidently characterized.
- Effective management requires collaboration among radiology, surgery, and pathology teams.

## Author contributions

All authors contributed significantly to this article. Saeed Mohammadzadeh: primary writer of manuscript. Alisa Mohebbi: providing revision for writing of manuscript. Afshin Mohammadi: imaging data interpretation. Ata Abbasi: data collection. Rahim Mahmodlou: manager and principal investigator, operation.

## CARE checklist statement

The guidelines of the CARE Checklist have been adopted.
